# A Comparison of Clinical Features between Community-Associated and Healthcare-Associated Methicillin-Resistant *Staphylococcus aureus* Keratitis

**DOI:** 10.1155/2015/923941

**Published:** 2015-01-12

**Authors:** Ching-Hsi Hsiao, Sherine Jue Ong, Chih-Chun Chuang, David H. K. Ma, Yhu-Chering Huang

**Affiliations:** ^1^Department of Ophthalmology, Chang Gung Memorial Hospital, Linkou 33305, Taiwan; ^2^College of Medicine, Chang Gung University, Taoyuan 33302, Taiwan; ^3^Department of Ophthalmology, Chang Gung Memorial Hospital, Keelung 20448, Taiwan; ^4^Department of Ophthalmology, Changhua Christian Hospital, Changhua 50006, Taiwan; ^5^Division of Pediatric Infectious Diseases, Department of Pediatrics, Chang Gung Memorial Hospital, Linkou 33305, Taiwan

## Abstract

*Purpose*. To compare the clinical features of community-associated (CA) and healthcare-associated (HA) methicillin-resistant *Staphylococcus aureus* (MRSA) keratitis. *Methods*. Patients presenting with culture-proven MRSA keratitis between January 1, 2006, and December 31, 2010, at Chang Gung Memorial Hospital, Taiwan, were included in this study. The patients' demographic and clinical information were reviewed retrospectively. Antibiotic susceptibility was verified using the disk diffusion method. *Results*. Information on 26 patients with MRSA keratitis was collected, including 12 cases of CA-MRSA and 14 cases of HA-MRSA. All MRSA isolates were susceptible to vancomycin; the only difference in drug susceptibility was that CA-MRSA isolates were more susceptible to trimethoprim/sulfamethoxazole than HA-MRSA (*P* = .034). The most common risk factor for MRSA keratitis was ocular surface disease. No significant differences were observed between the 2 groups in terms of clinical features, treatments, and visual outcomes. *Conclusion*. In Taiwan, CA-MRSA rivals HA-MRSA as a critical cause of MRSA keratitis. Furthermore, CA-MRSA isolates are multidrug resistant. CA-MRSA and HA-MRSA keratitis are clinically indistinguishable, although larger studies are warranted to further evaluate this association.

## 1. Introduction

*Staphylococcus aureus* is among the most important and commonly isolated human bacterial pathogens.* S. aureus* isolates resistant to methicillin, termed methicillin-resistant* S. aureus* (MRSA), are usually also resistant to other *β*-lactam antimicrobial drugs. MRSA, first identified in the 1960s, was traditionally associated with healthcare facilities, but its prevalence has reportedly increased in otherwise healthy patients without identified risk factors. Such infections are called community-associated MRSA (CA-MRSA), and they are clinically, microbiologically, and genetically distinct from healthcare-associated MRSA (HA-MRSA) [1]. CA-MRSA strains primarily involve infection of the skin and soft tissues, and they occasionally cause severe diseases [2]. They are frequently susceptible to other antistaphylococcal agents and carry genes for Panton-Valentine leukocidin (PVL) and may present a new acquisition of type IV or V staphylococcal cassette chromosome* mec* (SCC*mec*) DNA [1, 3, 4]. Recent clinical studies have further shown that the emerging MRSA strains were prevalent in the community and have begun replacing other MRSA strains in some hospitals [5]. CA-MRSA strains have emerged rapidly worldwide, becoming a critical public health problem [6]. Theoretically, CA-MRSA may have a distinct impact on patient outcomes in comparison with HA-MRSA, because most CA-MRSA isolates have special intrinsic virulence factors, such as PVL genes, and less resistance to antibiotics. However, previous studies specifically addressing the disease impacts of CA-MRSA and HA-MRSA, particularly on bacteremia, have reported conflicting results [7–11].

Until recently, few studies have focused on distinguishing between CA-MRSA and HA-MRSA strains in ocular MRSA infections [12–18]. In our previous 10-year study of MRSA ocular infections, we found that the most common ocular diseases caused by MRSA were keratitis, followed by lid disorder and conjunctivitis [19]. Compared with HA-MRSA, CA-MRSA mainly presented with lid and lacrimal system disorders and caused less vision-threatening disorders (i.e., keratitis, orbital cellulitis, or endophthalmitis) [14]. In addition, we analyzed 5-year results of* S. aureus* keratitis but did not observe the difference in clinical outcomes between methicillin-sensitive* S. aureus* (MSSA) and MRSA keratitis [20]; however, the difference in clinical outcomes between methicillin sensitive* S. aureus* (MSSA) and MRSA keratitis may have been minimized by the combination of CA-MRSA and HA-MRSA.

In this study, we divided MRSA isolates into CA-MRSA and HA-MRSA to compare the clinical features of CA-MRSA keratitis and HA-MRSA keratitis. Additionally, we sought to determine whether keratitis caused by the different MRSA isolates had distinct outcomes.

## 2. Materials and Methods

This clinical study was conducted in accordance with the Declaration of Helsinki, and approval was obtained from the Institutional Review Board of Chang Gung Memorial Hospital (CGMH). We accessed a computer database at the Microbiology Laboratory in CGMH and reviewed the corresponding medical records to identify both inpatients and outpatients with MRSA keratitis between January 1, 2006, and December 31, 2010, retrospectively.

The collected data included the patients' demographic information, medical and ocular history, presented signs and symptoms, systemic and local predisposing factors, presented visual acuity, antibiotic susceptibility, treatment, length of follow-up, and final visual acuity. The size and location of corneal infiltrates and the presence of hypopyon were documented at initial presentation. We determined the susceptibility of the isolates to 7 antibiotics (clindamycin, erythromycin, oxacillin, penicillin, trimethoprim/sulfamethoxazole, teicoplanin, and vancomycin) by using the disk diffusion method in accordance with the Clinical and Laboratory Standard Institute (CLSI) standards for antimicrobial susceptibility testing. We used oxacillin or cefoxitin to test for *β*-lactam antibiotic resistance. The definitions proposed by Naimi et al. [4] were used to define HA-MRSA and CA-MRSA in this study. HA-MRSA cases were defined as follows: (1) an MRSA infection identified after 48 hours of hospital admission; (2) a history of hospitalization, surgery, dialysis, or residence in a long-term care facility within 1 year of the MRSA culture date; (3) a permanent indwelling catheter or percutaneous medical device present at the time of culture; or (4) a known positive culture for MRSA before the study period. All other cases not matching these features were defined as CA-MRSA cases.

To treat MRSA keratitis, empirical or fortified antibiotics were administered hourly. The standard fortified antibiotics used in the study were topical amikacin (25 mg/mL), cefazolin sodium (25 mg/mL), or vancomycin (25 mg/mL), whereas the commercially available antibiotics used were topical fluoroquinolones (ciprofloxacin 0.3% or levofloxacin 0.5%). Subsequent modifications to the antibiotic treatment regimens were made according to the culture results, susceptibility testing, and clinical response. Surgical interventions, such as amniotic membrane transplantation, tarsorrhaphy, patch graft, or therapeutic penetrating keratoplasty, were indicated in some cases based on the clinical conditions. The healing time was also recorded once the infiltration had subsided and the epithelial defect had healed. Where data were available, visual acuity was recorded at least 2 months after the keratitis had subsided and stabilized. For the purpose of statistical analysis, Snellen visual acuity values were converted into logMAR units. The schedule reported by Scott et al. [21] was adopted to record the patients' visual acuity of counting fingers (CF), hand movements (HM), light perception (LP), and no light perception (NLP) as logMAR units.

Genotyping analysis, including pulsed-field gel electrophoresis (PFGE) typing, SCC*mec* elements, and the detection of PVL genes, was performed on available MRSA isolates. PFGE was used to fingerprint the MRSA clinical isolates according to the procedure described in a previous study [22]. The criteria proposed by Tenover et al. [23] were used to analyze the DNA fingerprints generated by the PFGE typing. The SCC*mec* typing was determined using a previously described multiplex polymerase chain reaction (PCR) strategy [24]. Control strains for SCC*mec* types I, II, III, and IVa (kindly provided by Dr. K. Hiramatsu) were as follows: type I, NCTC10442; type II, N315; type III, 85/2082; and type IVa, JCSC4744. The PCR amplification of the* lukS-PV* and* lukF-PV* genes encoding PVL components was described in a previous study [25].

Statistical analyses were performed using SPSS Version 17 (IBM, Armonk, NY, USA). The chi-square test or Fisher's exact test was used to analyze the nominal variables, and the Mann-Whitney *U* test was used for the continuous variables. Statistical significance was defined as *P* < .05.

## 3. Results

### 3.1. Demographics

Twenty-six cases of MRSA keratitis were identified, including 12 (46.2%) caused by CA-MRSA and 14 (53.8%) caused by HA-MRSA.  Table 1 shows the demographic data of the patients with MRSA keratitis. The rate of systemic comorbidities in the HA-MRSA group was significantly higher than in the CA-MRSA group (85.7% versus 41.7%, *P* = .038).

### 3.2. Clinical Findings

Table 2 presents the clinical findings of MRSA keratitis. The most common feature of keratitis, caused by either CA-MRSA or HA-MRSA, was central and medium-sized corneal ulcers. There was no difference in the location, infiltration size, and presence of hypopyon between the 2 groups.

### 3.3. Predisposing Factors

Table 3 lists the predisposing factors for MRSA keratitis. The most common predisposing factor for both CA-MRSA and HA-MSSA keratitis was ocular surface disease, which was accounted for 21 (80.7%) of the MRSA keratitis cases. Additionally, a higher percentage of the patients with HA-MRSA keratitis had a recent history of using topical antibiotics or corticosteroids (*P* = .014). No difference was observed between the 2 groups for other local risk factors, such as contact lens wear, trauma, and previous ocular surgery.

### 3.4. Antibiotic Susceptibility

Table 4 lists the antibiotic susceptibility of MRSA. All MRSA isolates were susceptible to vancomycin and teicoplanin, but resistant to clindamycin, erythromycin, and penicillin. Compared with the HA-MRSA isolates, the CA-MRSA isolates were significantly more susceptible to sulfamethoxazole/trimethoprim (83.3% versus 42.9%, *P* = .034).

### 3.5. Treatment and Outcome

Table 5 presents treatment and outcome of MRSA keratitis. All patients with MRSA keratitis were treated with topical antibiotics. A combination of fortified antibiotics (cefazolin sodium 25 mg/mL and amikacin 25 mg/mL) or fluoroquinolone alone (ciprofloxacin 0.3% or levofloxacin 0.5%) was the most commonly used initial empirical treatment. In 17 cases, the medication regimen was shifted to vancomycin 25 mg/mL after obtaining culture results, and the rate of modification did not differ significantly between the 2 groups (*P* = .683). Five patients with CA-MRSA keratitis and 4 patients with HA-MRSA keratitis required surgical intervention. Three patients were refractory to medical treatment; 2 patients (one in each group) underwent patch grafts with glycerol-preserved cornea; one patient with severe CA-MRSA keratitis, who had no light perception at presentation and responded poorly to medical treatment, underwent evisceration. Other 6 patients received amniotic membrane transplantation or tarsorrhaphy to promote reepithelialization. There were no significant differences in the rate of surgical intervention, admission, severe complications (including corneal perforation and endophthalmitis), or healing time between the 2 groups. In addition, there was no significant difference between the 2 groups in terms of the final visual outcomes.

### 3.6. Genotyping Analysis

Eight MRSA isolates were available for the genotyping analysis. One of the HA-MRSA isolates (*n* = 2) was characterized as ST 5/PFGE type F/SCC*mec* II/PVL-negative, and the other was ST 239/PFGE type A/SCC*mec* IIIA/PVL-negative, both of which were compatible with those of HA-MRSA isolates reported in our previous pediatric study [26]. Five of the 6 CA-MRSA isolates were characterized as ST 59/PFGE type C/SCC*mec* IV/PVL-negative and the other was ST 59/PFGE type D/SCC*mec *V_T_/PVL-positive. Both clones shared the common genetic characteristics of CA-MRSA strains in Taiwan [26].

## 4. Discussion

A PubMed search revealed no previous study focusing on the clinical profiles of MRSA keratitis, including demographics, predisposing factors, clinical manifestation, drug susceptibility, treatments, and outcomes, specifically comparing CA-MRSA and HA-MRSA. Our findings show that 46% of MRSA keratitis cases were caused by CA-MRSA. Both of the MRSA strains were resistant to multiple antibiotics, and the differences in visual outcomes between the CA-MRSA and HA-MRSA keratitis cases were nonsignificant.

No significant difference in clinical manifestations was observed between the CA-MRSA and HA-MRSA keratitis cases in this study. Shanmuganathan et al. [27] and Freidlin et al. [28] reported several cases of MRSA keratitis, and they described that MRSA keratitis was usually nondestructive and nonthreatening to vision. Khan et al. [17] reported 6 cases of keratitis caused by MRSA isolates (5 SCC*mec* II isolates and one SCC*mec* III isolate, which belonged to HA-MRSA), all of which had minimal superficial defects and subepithelial infiltrates. In our series, most patients had medium-sized infiltrates, and 3 CA-MRSA patients and one HA-MRSA patient had corneal perforation or endophthalmitis. Compared with previously reported cases of MRSA keratitis, the infections observed in our study appeared to be more severe.

In this study, the most common local predisposing factor for both CA-MRSA and HA-MRSA keratitis was ocular surface disease; more patients with HA-MRSA keratitis had a recent history of using topical antibiotics or corticosteroids. Previous reports indicated that ocular surface disease was a significant risk factor for MRSA keratitis [27–29]. Hori et al. [30] found that 1% of preoperative patients carried MRSA on the conjunctiva. Fukuda et al. [31] reported that MRSA can appear as normal conjunctival flora in as many as 10.3% of elderly patients. In addition, local immunocompromised status could promote colonized MRSA to be pathogenic. In our patients with MRSA keratitis, the associated ocular surface diseases included dry eye, exposure keratitis, trichiasis, Stevens-Johnson syndrome, and ocular pemphigoid. Such patients frequently had a compromised integrity of ocular surface and corneal epithelial defect; they were typically treated with topical corticosteroids and antibiotics, and they occasionally wore therapeutic contact lenses. All of these factors might predispose the patients to MRSA corneal infection. The higher rate of systemic comorbidities and previous use of topical antibiotics or corticosteroids in the patients with HA-MRSA keratitis in this study was probably because these patients might require frequent hospital visits; hence, they were more exposed to HA-MRSA.

The antibiograms of CA-MRSA isolates in this study differed from those identified in the United States, which showed that CA-MRSA strains were typically susceptible to a wide range of non-*β*-lactam antibiotics, such as clindamycin [4, 16]. We also found that CA-MRSA as well as HA-MRSA isolates were multidrug resistant, and susceptibility differed only for trimethoprim/sulfamethoxazole, which was comparable to the reports in the nonocular field in Taiwan [5, 14]. Two national surveys of ocular isolates conducted in the United States reported a high rate of resistance (>80%) to fluoroquinolones in MRSA [32, 33]. Recently, Hong et al. [34] reported that in, China, CA-MRSA was more susceptible to fluoroquinolones than HA-MRSA. We did not test fluoroquinolones in our microbiology laboratory because they were not included in the recommended list of antibiotics published by the CLSI. Because fluoroquinolones are effective broad-spectrum antibiotics and are commonly used as an empirical monotherapy for bacterial keratitis, we would extend the antibiotic susceptibility profiles to include fluoroquinolones to determine whether there are any differences between CA-MRSA and HA-MRSA isolates in future studies.

Although the rate of admission, complications, and surgical intervention was higher in the patients with CA-MRSA keratitis, there was no statistically significant difference between the 2 groups. Additionally, we did not detect a difference in the visual outcomes between the 2 groups. Most CA-MRSA isolates have some virulence factors, such as PVL genes, so it is plausible that infection caused by CA-MRSA could lead to worse outcomes than those caused by HA-MRSA. Conversely, CA-MRSA isolates are more susceptible than HA-MRSA isolates to various antibiotics; thus, infections caused by CA-MRSA should be easier to handle. Several studies comparing the difference in clinical outcomes between CA-MRSA and HA-MRSA bacteremia have reported conflicting results [7–9, 11], which are probably attributable to study design or quality, or the definition of clinical outcomes and CA-MRSA. Few ocular studies have distinguished between CA-MRSA and HA-MRSA strains. Rutar et al. [13] reported 9 patients with CA-MRSA (USA300) infections manifested as orbital cellulitis, endogenous endophthalmitis, panophthalmitis, lid abscesses, and septic venous thrombosis, but there was no mention of keratitis. The authors of that study acknowledged that severe MRSA infections may have been overrepresented because only isolates from cultures were included in that study. Sueke et al. [35] compared the clinical outcomes of keratitis caused by PVL-positive and -negative* S. aureus* and found that PVL-positive isolates were associated with a trend of poorer clinical outcomes and more frequent surgical interventions, indicating that PVL may be a critical virulence factor in* S. aureus* keratitis. There are 2 prevalent CA-MRSA clones in Taiwan: ST 59/PFGE type C/SCC*mec* IV/PVL-negative and ST 59/PFGE type D/SCC*mec *V_T_/PVL-positive [26]. Our clinical microbiology laboratories retain only isolates from blood for long-term storage; only 8 isolates were available for genotyping analysis, so it was difficult for us to determine whether there were interspecies differences in the CA-MRSA keratitis cases observed in this study. Moreover, the statistical power of our study was limited by the small sample size. Further study with a larger sample is warranted to determine whether patients with CA-MRSA and HA-MRSA keratitis have different clinical outcomes.

This study was limited by its retrospective nature and small sample. The treatment protocols varied among the physicians, and there were inherent flaws of visual acuity with variable interval as an outcome measure. In addition, we used oxacillin testing as a surrogate to identify the resistant species of* S. aureus*, and we defined CA-MRSA and HA-MRSA according to the epidemiological differences, not according to genetic characterization. Moreover, the patient selection criteria may have influenced the data interpretation because our patients came from a referral-based tertiary-care institution. Therefore, caution should be applied when generalizing the results.

## 5. Conclusion

In Taiwan, CA-MRSA rivals HA-MRSA as a key cause of MRSA keratitis. CA-MRSA isolates are multidrug resistant, but more susceptible to trimethoprim/sulfamethoxazole than HA-MRSA isolates. CA-MRSA and HA-MRSA keratitis are clinically indistinguishable, although they have distinct phenotypic and molecular characteristics. Nevertheless, larger studies are warranted to further evaluate this association.

## Figures and Tables

**Table 1:** Comparison of demographics and characteristics of the patients with community-associated methicillin-resistant* Staphylococcus aureus* (CA-MRSA) and healthcare-associated methicillin-resistant *Staphylococcus aureus *(HA-MRSA) keratitis.

Characteristics	CA-MRSA (*n* = 12)	HA-MRSA (*n* = 14)	*P* value
Age: median (range)	59.5 (8~83)	51.0 (2~82)	.297
Gender: M/F	5/7	10/4	.126
Eye: R/L/B	3/9/0	6/7/1	.322^*^
Systemic comorbidities: *n* (%)	5 (41.7)	12 (85.7)	.038
Use of immunosuppressants: *n* (%)	0 (0)	1 (7.1)	1
Use of systemic antibiotics: *n* (%)	0 (0)	2 (14.3)	.483

Mann-Whitney test was used for age comparison and chi-square test for others.

^*^ *P* value obtained by Fisher's exact test.

M: male, F: female. R: right eye, L: left eye, and B: both eyes.

**Table 2:** Clinical findings of community-associated methicillin-resistant* Staphylococcus aureus* (CA-MRSA) and healthcare-associated methicillin-resistant *Staphylococcus aureus *(HA-MRSA) keratitis.

Findings	CA-MRSA (*n* = 12) *N* (%)	HA-MRSA (*n* = 14) *N* (%)	*P *value
Location			.856
Central	8 (66.7)	8 (57.1)	
Paracentral	2 (16.7)	4 (28.6)	
Peripheral	2 (16.7)	2 (14.3)	
Infiltration size (mm)			1
Small (<2)	2 (16.7)	1 (7.7)	
Medium (2~6)	9 (75.0)	10 (76.9)	
Large (>6)	1 (8.3)	2 (15.4)	
Hypopyon	1 (8.3)	4 (28.6)	.330

*P* value was obtained by Fisher's exact test.

**Table 3:** Predisposing factors for community-associated (CA) methicillin-resistant* Staphylococcus aureus* and healthcare-associated (HA) methicillin-resistant *Staphylococcus aureus *keratitis.

Predisposing factors	CA-MRSA (*n* = 12) *N*, %^*^	HA-MRSA (*n* = 14) *N*, %^*^	*P* value
Contact lens wear	2 (16.7)	2 (14.3)	1
Trauma	1 (8.3)	0 (0)	.462
Ocular surface disease	10 (83.3)	11 (78.6)	1
Previous ocular surgery	3 (25)	7 (50)	.248
Use of topical antibiotics/immunosuppressant	1 (8.3)	8 (57.1)	.014

*P* value was obtained by Fisher's exact test.

^*^Total is greater than 100% because some patients had multiple risk factors.

**Table 4:** Antibiotic susceptibility of community-associated methicillin-resistant* Staphylococcus aureus* (CA-MRSA) and healthcare-associated methicillin-resistant *Staphylococcus aureus* (HA-MRSA) isolates.

Antibiotics	CA-MRSA (*n* = 12) *N* (%)	HA-MRSA (*n* = 14) *N* (%)	*P* value
Clindamycin	0 (0)	0 (0)	
Erythromycin	0 (0)	0 (0)	
Penicillin	0 (0)	0 (0)	
Sulfa-Trim	10 (83.3)	6 (42.9)	.034
Teicoplanin	12 (100)	14 (100)	
Vancomycin	12 (100)	14 (100)	

Sulfa-Trim: sulfamethoxazole/trimethoprim.

*P* value was obtained by Chi-sqaure test.

**Table 5:** Treatment and clinical outcome of community-associated methicillin-resistant* Staphylococcus aureus* (CA-MRSA) and healthcare-associated methicillin-resistant *Staphylococcus aureus* (HA-MRSA) keratitis.

	CA-MRSA (*n* = 12) *N* (%)	HA-MRSA (*n* = 14) *N* (%)	*P* value
Modification of antibiotics: *n* (%)	9 (81.8)	8 (61.5)	.386^*^
Surgical intervention: *n* (%)	5 (41.7)	4 (28.6)	.683^*^
Admission: *n* (%)	8 (66.7)	5 (35.7)	.253^*^
Severe complication^†^: *n* (%)	3 (25)	1 (7.1)	.306^*^
Complete remission^‡^ (months): median (mini~max)	1.335 (0.2~8.2)	0.93 (0.2~8.5)	.348
VA (logMAR)^#^: median (mini~max)			
Initial	3 (0~3)	3 (0~3)	.331
Final	1.7 (0~3.2)	3 (0.4~3.2)	.282

Mann-Whitney test was used for comparison of VA and complete remission and chi-square test for others.

^*^ *P* value obtained by Fisher's exact test.

^†^Severe complication: corneal perforation and/or endophthalmitis.

^‡^Complete remission: defined as the resolution of infiltration and epithelial defect.

^#^VA: visual acuity, recorded using Snellen letter charts. If data were available, Snellen VA was converted to logarithm of the minimum angle (logMAR).
